# Noninvasive Management of Fractured Indwelling Tunneled Pleural Catheter Valve

**DOI:** 10.1155/2022/2541285

**Published:** 2022-08-13

**Authors:** Azib Shahid, Harpreet Singh, Toni-Denise Espina, Mohammad Abdalla, Uzair Ghori, Bryan S. Benn

**Affiliations:** ^1^Department of Medicine, Rosalind Franklin University of Medicine and Science, Chicago, Illinois, USA; ^2^Department of Pulmonary and Critical Care Department, Medical College of Wisconsin, Milwaukee, WI, USA

## Abstract

Tunneled indwelling pleural catheters (IPCs) are frequently used to palliate symptomatic dyspnea due to recurrent pleural effusions. The drainage valve of IPCs is an important component of the catheter as fracture of the valve leads to malfunctioning of the IPCs. Replacement of the catheter includes risks such as pain, infection, pneumothorax, and procedure cost. We report two cases of malfunctioning tunneled IPC drainage valves repaired by our noninvasive method and discuss the need for a repair kit and a standardized approach to this repair in case of nonavailability of repair kits.

## 1. Introduction

Tunneled indwelling pleural catheters (IPCs) are an appropriate choice to palliate symptomatic dyspnea due to malignant or recurrent pleural effusions [1–3]. IPCs are silicon tubes, tunneled and secured subcutaneously using a profibrotic polyester cuff, with a self-sealing, one-way valve as the external end piece [4].

The drainage valve is an important component of the catheter because it remains closed until accessed by the draining line [2–4]. Fracture of the valve leads to passage of air or fluid through the catheter into the pleural cavity, potentially resulting in hydropneumothorax [5, 6]. Although malfunctioning rarely occurs [4], IPCs are being used more frequently [4]. Risks associated with IPC replacement include pain, infection, pneumothorax, and cost [4, 7]. Thus, attempts to fix a malfunctioning drainage valve are reasonable, although no standard approach exists. We report two cases of malfunctioning IPC drainage valves repaired by our noninvasive method and discuss the need for a standardized approach to this process.

## 2. Case Presentations

### 2.1. Case 1

A 73-year-old man with metastatic left clear cell renal cell carcinoma with a recurrent left malignant pleural effusion had an IPC placed for symptomatic dyspnea. About 1 year later, he presented with dyspnea on exertion and tachycardia. His IPC was draining approximately 50 cc/day, whereas previously, it drained about 500 cc/day. He was found to be hemodynamically unstable and subsequently fluid resuscitated. Oxygen saturation was >94% on room air. Chest computed tomography (CT) scan showed a moderate-sized left pleural effusion. No pneumothorax was reported. The IPC valve was inspected and found to have a fracture of the plastic hub surrounding the valve. The catheter insertion site was clean, dry, and intact.

### 2.2. Case 2

A 62-year-old man with a recurrent right pleural effusion due to end-stage renal disease and congestive heart failure with reduced ejection fraction had a right IPC placed for symptomatic relief. Approximately 8 months later, he presented to the hospital with dyspnea and decreased drainage from his IPC. He reported that he had misplaced the drainage valve cap and was having difficulty with insertion of the drainage access tip into the valve. Vitals were stable. Chest X-ray showed a moderate right pleural effusion. No pneumothorax was reported. The catheter insertion site was clean, dry, and intact.

**Figure 1:**
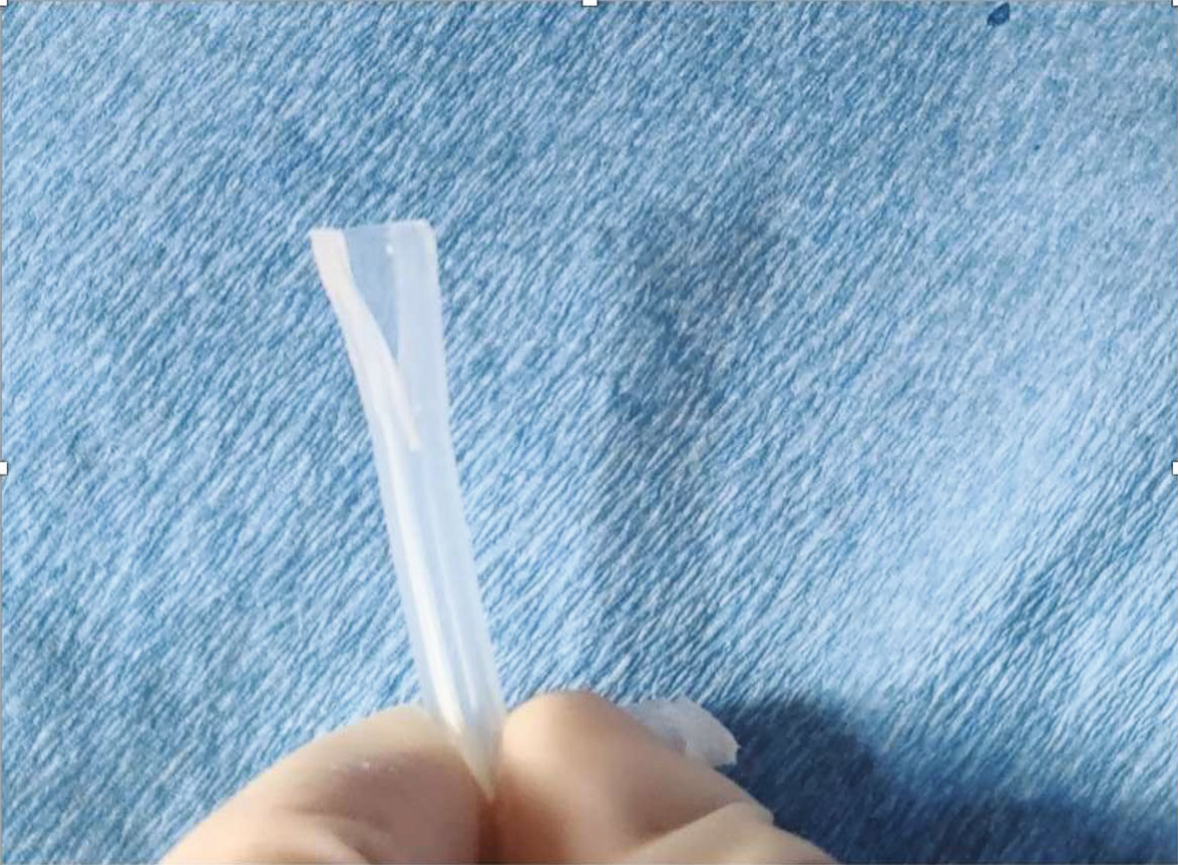
Longitudinal incision made on the distal end of the new indwelling pleural catheter tip to free the drainage valve.

**Figure 2:**
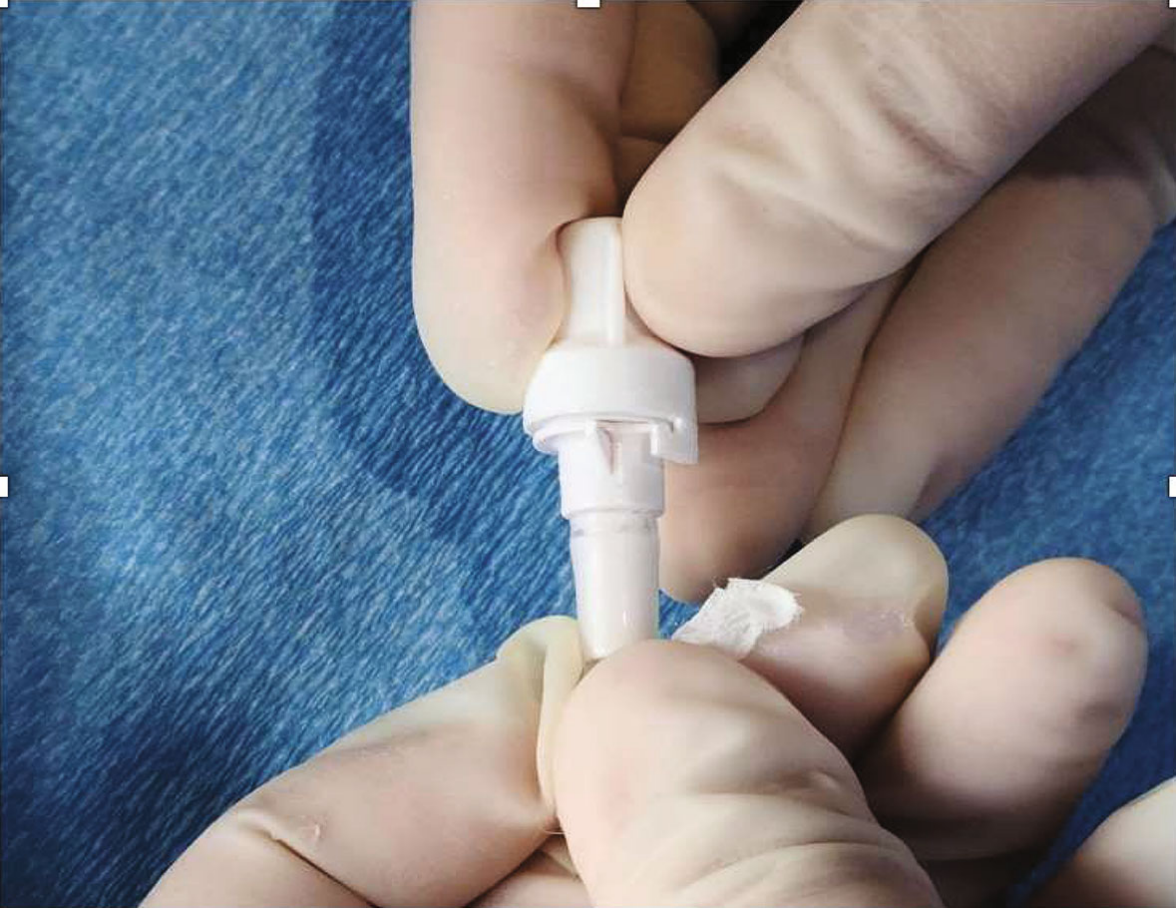
Cyanoacrylate tissue adhesive applied to the tapering end of the new drainage valve and inserted into the old catheter.

**Figure 3:**
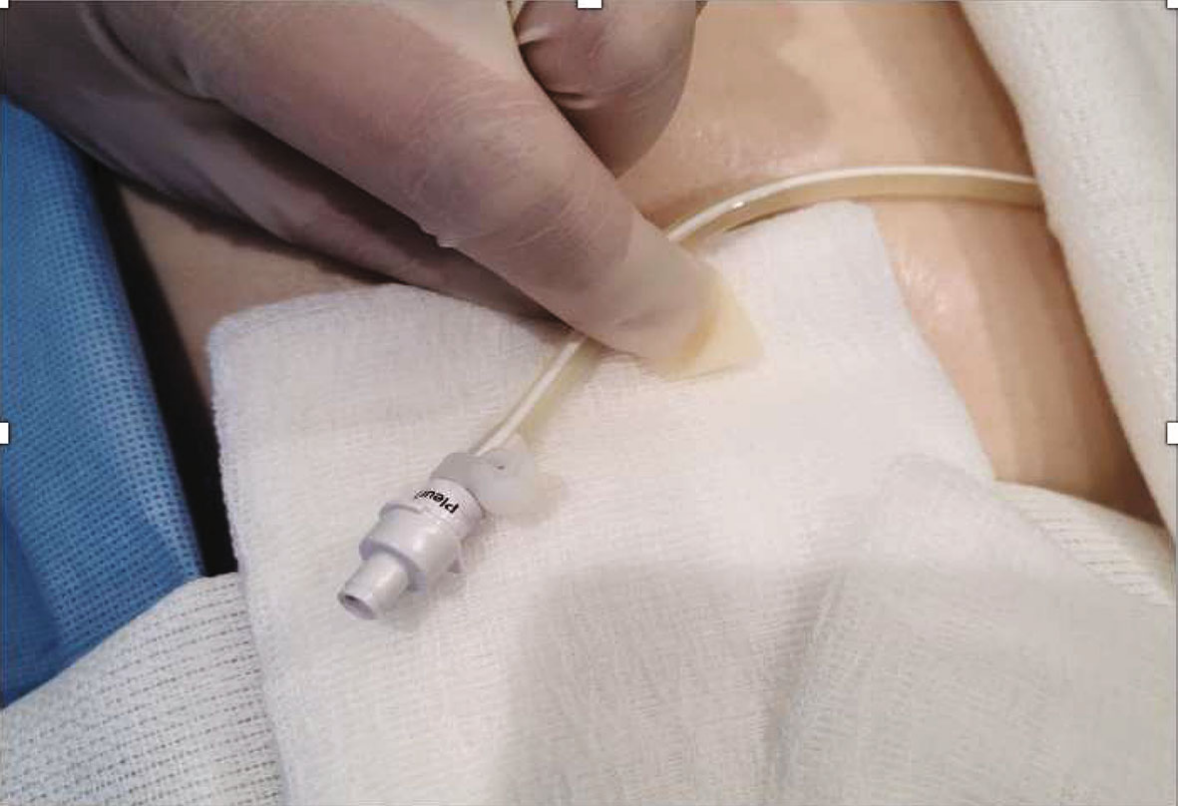
New indwelling pleural catheter drainage valve tightened at the end of the old indwelling pleural catheter with a zip tie.

Risks and benefits of replacing the drainage valve versus replacing the entire IPC were discussed, and both patients opted to replace the valve alone. Under sterile conditions, the IPC was clamped, and the fractured valve was removed by cutting the catheter distal to the clamp. A new pleural catheter drainage valve was obtained from a pleural catheter drainage kit by making a longitudinal incision at the distal end of the catheter tubing fitted onto the tapered end of the drainage valve (Figure 1). Cyanoacrylate tissue adhesive (adhesive used to form a strong bond between apposed wound edges) was applied on the new catheter valve, which was inserted into the exposed end of the old catheter till its base (Figure 2) and tightened using a zip tie (Figure 3). The catheter was then unclamped, and adequate drainage of fluid was seen, which improved both patients' symptomatic dyspnea.

In case 1, immediately after repair, 500 ml of straw-colored pleural fluid was drained. In case 2, approximately 600 ml of pleural fluid was drained. Upon long-term follow-up, no recurrent leaks or dislocation occurred. The patients did not experience any infectious complications attributed to catheter repair or manipulation. Case 2 passed away 7 months later due to cardiovascular disease.

## 3. Discussion

In the United States, two different types of IPCs are mostly used: PleurX catheter (BD) or Aspira (Bard) catheter. The former uses vacuum bottles, while the latter uses a manual pump to facilitate fluid collection. Both systems have shown a similar outcome and safety profile [8]. In clinical practice, both catheters are used, but most studies comparing IPCs with chemical pleurodesis have used the PleurX catheter [1–3]. Aspira has had a separate valve repair kit, while PleurX replacement parts have become available only recently [9]. In the event of an IPC valve fracture or separation from the catheter, a new IPC can be placed, but this involves risk to the patient [3]. The IPC has a cuff which anchors it into the subcutaneous tissue by forming fibrinous adhesions. Removal of the IPC requires significant traction force to disengage the anchoring cuff, requiring iatrogenic severing [6]. It would also subject the patient to another invasive procedure when putting in a new IPC with potential associated complications, including infection, pain, or pneumothorax [4].

Bower and Mahmood described two scenarios: (1) fracture of PleurX valve and (2) separation of PleurX valve from the catheter. In the first case, the PleurX valve was replaced with an Aspira valve replacement kit, so the patient had to use a manual pump with an Aspira drainage bag. In the second case, since valve integrity was intact, the same valve was reattached using a sterile tie strap after cutting 2 cm off the end of the catheter [9].

Knox and Rollins reported a weakness in the PleurX system which led to separation of the PleurX valve from the catheter. In contrast to the second scenario above described by Bower and Mahmood, this case was complicated with a pleural space infection. Therefore, another PleurX kit was opened to obtain a new valve which was fixed onto the existing tubing using cyanoacrylate [10].

Morris and Schwartz reported fracture of the PleurX valve hub required to secure the valve cap. Given the risk of infections, they replaced the valve with a new one obtained from a new PleurX kit and secured it into place using cyanoacrylate after cutting 2 cm off the distal end of the catheter [7].

All cases report the importance of a dry internal surface to ensure stability of the repair as moisture leads to instability and the valve potentially sliding out of the IPC. Since no standard valve repair kits were available from the manufacturer at that time, these repairs used various adhesives available at the respective facilities to attach the valve to the IPC. All patients were reported to have good outcomes [7, 9, 10].

IPCs have significantly improved the quality of life of patients with symptomatic pleural effusions [3]. These devices are used frequently for fluid collection, and handling of the valve can sometimes lead to damage [6, 9, 10]. Although this complication is rare, it may happen more due to the sheer volume of procedures going up as more indications become available, for example, as a bridge to transplant in end-stage renal disease, congestive heart failure, and liver failure [4]. In these situations, a standardized approach of noninvasive repair of the drainage system is important. Removing the valve from a new catheter increases supply cost. A standard repair kit recently introduced by PleurX will make the process more convenient, economic, and efficient. Standardizing the approach will provide a structured method and reliable data and will save time in the innovation process. It will also build the patient's trust in the product and the services of the physician.

## Data Availability

Data will be made available upon request.
